# A Valuable Series

**Published:** 1916-05

**Authors:** 


### A Valuable Series.

Much attention is being directed to the series of articles by Dr. Harrower beginning in this issue of the Texas Medical Journal and to be continued for ten or twelve months. Perhaps no more valuable thing has ever been done for the practicing physician than the service thus rendered them by Dr. Harrower and the Texas Medical Journal. A number of new subscriptions have been received in anticipation of these valuable articles, and we confidently expect many more as soon as physicians generally learn what is being offered them. Do not lose this issue, but place it away carefully after you have read it and keep the file until you have had the entire series of articles. It is probable that you will want to bind them for ready use in future.

